# Organic Copper Speciation by Anodic Stripping Voltammetry in Estuarine Waters With High Dissolved Organic Matter

**DOI:** 10.3389/fchem.2020.628749

**Published:** 2021-02-02

**Authors:** Jasmin Pađan, Saša Marcinek, Ana-Marija Cindrić, Chiara Santinelli, Simona Retelletti Brogi, Olivier Radakovitch, Cédric Garnier, Dario Omanović

**Affiliations:** ^1^ Rud−er Bošković Institute, Center for Marine and Environmental Research, Zagreb, Croatia; ^2^ CNR-Biophysics Institute, Pisa, Italy; ^3^ CNRS, IRD, INRAE, Coll France, CEREGE, Aix-Marseille University, Marseille, France; ^4^ IRSN (Institut de Radioprotection et de Sûreté Nucléaire), PSE-ENV/SRTE/LRTA, Saint-Paul-Les-Durance, France; ^5^ Mediterranean Institute of Oceanology, ECEM, Toulon University, La Garde, France

**Keywords:** Arno River estuary, copper, organic ligands concentration, speciation, trace metals, surface active substances, metal complexing capacity

## Abstract

The determination of copper (Cu) speciation and its bioavailability in natural waters is an important issue due to its specific role as an essential micronutrient but also a toxic element at elevated concentrations. Here, we report an improved anodic stripping voltammetry (ASV) method for organic Cu speciation, intended to eliminate the important problem of surface-active substances (SAS) interference on the voltammetric signal, hindering measurements in samples with high organic matter concentration. The method relies on the addition of nonionic surfactant Triton-X-100 (T-X-100) at a concentration of 1 mg L^−1^. T-X-100 competitively inhibits the adsorption of SAS on the Hg electrode, consequently 1) diminishing SAS influence during the deposition step and 2) strongly improving the shape of the stripping Cu peak by eliminating the high background current due to the adsorbed SAS, making the extraction of Cu peak intensities much more convenient. Performed tests revealed that the addition of T-X-100, in the concentration used here, does not have any influence on the determination of Cu complexation parameters and thus is considered "interference-free." The method was tested using fulvic acid as a model of natural organic matter and applied for the determination of Cu speciation in samples collected in the Arno River estuary (Italy) (in spring and summer), characterized by a high dissolved organic carbon (DOC) concentration (up to 5.2 mgC L^−1^) and anthropogenic Cu input during the tourist season (up to 48 nM of total dissolved Cu). In all the samples, two classes of ligands (denoted as L_1_ and L_2_) were determined in concentrations ranging from 3.5 ± 2.9 to 63 ± 4 nM eq Cu for L_1_ and 17 ± 4 to 104 ± 7 nM eq Cu for L_2_, with stability constants log*K*_Cu,1_ = 9.6 ± 0.2–10.8 ± 0.6 and log*K*_Cu,2_ = 8.2 ± 0.3–9.0 ± 0.3. Different linear relationships between DOC and total ligand concentrations between the two seasons suggest a higher abundance of organic ligands in the DOM pool in spring, which is linked to a higher input of terrestrial humic substances into the estuary. This implies that terrestrial humic substances represent a significant pool of Cu-binding ligands in the Arno River estuary.

## Introduction

Copper (Cu) is an essential micronutrient in natural waters, required for the proper functioning of metabolic and respiratory processes for many aquatic species (Peers et al., 2005; Peers and Price, 2006; Annett et al., 2008; Glass and Orphan, 2012; Jacquot et al., 2014). It is also known for its toxic effects when exceeding a critical concentration threshold (Karlsson et al., 2010; Ytreberg et al., 2010) and thus is considered a specific pollutant of great ecotoxicological concern (Corcoll et al., 2019; Lopez et al., 2019). It is not the total copper concentration that is directly related to ecotoxicological effects but the fraction available for biological uptake (free and/or labile concentration) (Campbell et al., 2002; Sanchez-Marin et al., 2016; Zitoun et al., 2019). Therefore, for an accurate assessment of its potential impact on biota, the knowledge of Cu speciation, i.e., predicting the concentration of its bioavailable fraction, is of primary concern. The most important factor influencing Cu speciation in seawater is the concentration and quality (chemical structure) of the dissolved organic matter (DOM) (Sanchez-Marin et al., 2016). The range of copper concentrations between its necessity and toxicity is relatively narrow (Amin et al., 2013; Zitoun et al., 2019). However, the formation of strong complexes with organic ligands can reduce the bioavailable Cu fraction and, in most cases, maintains it in the optimal range (Buck et al., 2012; Whitby and van den Berg, 2015; Whitby et al., 2017). The presence of organic ligands is therefore of main significance in assessing the Cu bioavailability, with respect to both toxicity and necessity. Nevertheless, there is evidence that even organically complex copper is acquired by marine phytoplankton and bacteria (Semeniuk et al., 2015). Coastal areas and estuaries are the most relevant areas for Cu speciation studies because of the high potential for anthropogenic Cu contamination (e.g., Helland and Bakke, 2002; Blake et al., 2004; Louis et al., 2009) including secondary contamination by the release from sediment during estuarine mixing (e.g., Cobelo-Garcia and Prego, 2003). These areas are usually characterized by high concentrations of DOM from both allochthonous and autochthonous sources (Fellman et al., 2011; Retelletti Brogi et al., 2020), including biogenic thiol compounds and terrestrially derived humic and fulvic acids, which all form strong Cu complexes (Tang et al., 2000; Kogut and Voelker, 2001; Laglera and van den Berg, 2003; Shank et al., 2004; Whitby et al., 2017; Dulaquais et al., 2020). Besides regulating its bioavailable concentration, binding with these organic ligands in coastal waters is an important factor in Cu transport to the ocean (Helland and Bakke, 2002).

Due to experimental limitations of separation, extraction, and direct measurement of different metal-organic ligand complexes in seawater, an alternative approach based on complexometric titrations using electrochemical techniques (known as the determination of the metal complexing capacity of the sample) is preferentially used (Buck et al., 2012; Pižeta et al., 2015). As the concentration of metals in seawater is very low, various electrochemical techniques with low detection limits are commonly used, mainly stripping techniques: anodic and competitive ligand exchange adsorptive cathodic stripping voltammetry (ASV and CLE-AdCSV, respectively) (Sanchez-Marin et al., 2010; Buck et al., 2012; Omanović et al., 2015; Pižeta et al., 2015; Whitby et al., 2018). Knowledge about sources and chemical identity of detected ligands is scarce (Vraspir and Butler, 2009) and its acquisition is hindered by very complex chemical composition of natural DOM (Repeta, 2015). Useful qualitative information about the DOM pool in the aquatic environment (e.g., average aromaticity degree, molecular weight, and occurrence of humic-like, fulvic-like, and protein-like substances) can be gained indirectly via UV/Vis and fluorescence spectroscopic studies of its optically active fractions (colored and fluorescent dissolved organic matter, CDOM and FDOM) (Yamashita et al., 2011; Osburn et al., 2012; Lee et al., 2018; Galletti et al., 2019; Marcinek et al., 2020). Including this information in speciation studies could improve our understanding of the sources and composition of metal-binding organic ligands (Sanchez-Marin et al., 2010; Slagter et al., 2017; Watanabe et al., 2018; Wong et al., 2019; Whitby et al., 2020). However, these methods cannot characterize the binding sites or give information about nonfluorescent substances in DOM. This is possible by analyses of the molecular composition of DOM isolated from estuarine water (Minor et al., 2001; Minor et al., 2002; Dalzell et al., 2009; Abdulla et al., 2010; Zhang et al., 2018; Daoud and Tremblay, 2019; Thibault et al., 2019), e.g., using Fourier-transform infrared spectroscopy (FTIR) and nuclear magnetic resonance spectroscopy (NMR) (Abdulla et al., 2010; Zhang et al., 2018) or direct temperature-resolved mass spectrometry (DT-MS) complemented with either size-exclusion chromatography (Minor et al., 2002) or wet chemical techniques (Minor et al., 2001), which provide qualitative and quantitative analyses of major functional groups and information about their distribution.

Voltammetric stripping techniques are particularly sensitive to the composition of the sample solution. The well-known interferences in natural samples are due to the adsorption of surface-active substances (SAS) on the surface of the working electrode (usually Hg-drop) (Sander and Henze, 1996; Batley et al., 2004; Hurst and Bruland, 2005). This is obstructing both deposition/adsorption and stripping steps in ASV and CLE-AdCSV, resulting in lower sensitivity and/or deformation of the resultant voltammetric peak (Boussemart et al., 1993; Scarano and Bramanti, 1993; Plavšić et al., 1994; Louis et al., 2008). The extent to which SAS adsorbs on the electrode surface depends on the sample composition and the type of SAS. The final negative effect of the SAS influence is the inaccuracy of speciation parameters (Louis et al., 2008). Given that SAS is a significant fraction of DOM in seawater (Ciglenečki et al., 2020), the analysis of samples with high DOM content is particularly challenging. At potentials < −1.4 V, desorption of SAS from the electrode surface occurs (Sahlin and Jagner, 1996). Therefore, for removing the interferences during measurement, the "desorption step" (DS) was proposed (Louis et al., 2008; Louis et al., 2009; Gibbon-Walsh et al., 2012), i.e., switching to very negative potentials (e.g., −1.5 V) for a short time (e.g., 1–3 s), at the end of the main deposition period. DS reduces the effects of interferences to a large extent and enables a better electrode response and the formation of a well-defined peak. However, in samples with high DOM content, adsorption interferences cannot be completely removed, and some effects still exist. Previous studies showed that nonionic surfactant Triton-X-100 (T-X-100) added to the sample has an influence on the redox processes of Cu (Krznarić et al., 1994; Plavšić et al., 1994) and helps in obtaining reliable Cu complexation parameters in model solutions (Omanović et al., 1996). Based on this evidence, we assumed that its adsorption properties could be beneficial also for Cu speciation in natural samples hindered by the adsorbed SAS.

The main objective of this study is to explore the ability of nonionic surfactant T-X-100 to eliminate SAS interferences on the mercury electrode in the presence of high concentrations of DOM, without disturbing the original chemical speciation of Cu in the sample. The method was tested using fulvic acid (obtained from the International Humic Substances Society, IHSS) as a model of natural organic matter. The benefit of the proposed method for copper speciation studies is demonstrated in samples from the Arno River estuary (Italy), characterized by high organic matter content (up to 5.2 mg L^−1^ of dissolved organic carbon, DOC) and significant anthropogenic copper concentration (up to 48 nM of total dissolved copper, DCu). Obtained complexation parameters (i.e., detected Cu-binding ligands) are complemented with UV/Vis and fluorescence measurements (PARAFAC analysis) of DOM present in the estuary.

## Materials and Methods

### Study Area

The Arno River is 242 km long (the 5th largest river in Italy) and its catchment covers an area of 8,228 km^2^. The river flows into the Ligurian Sea about 10 km downstream from Pisa. The Arno River is impacted by various anthropogenic sources. Industrial activities like paper-mills, textile, electrochemical plants, and tanneries contribute to high levels of various inorganic and organic contaminants (Dinelli et al., 2005; Cortecci et al., 2009). For example, the Arno River accounts for 7% of the total DOC flux entering the western Mediterranean Sea, highlighting its relevant contribution (Retelletti Brogi et al., 2020).

**FIGURE 1:**
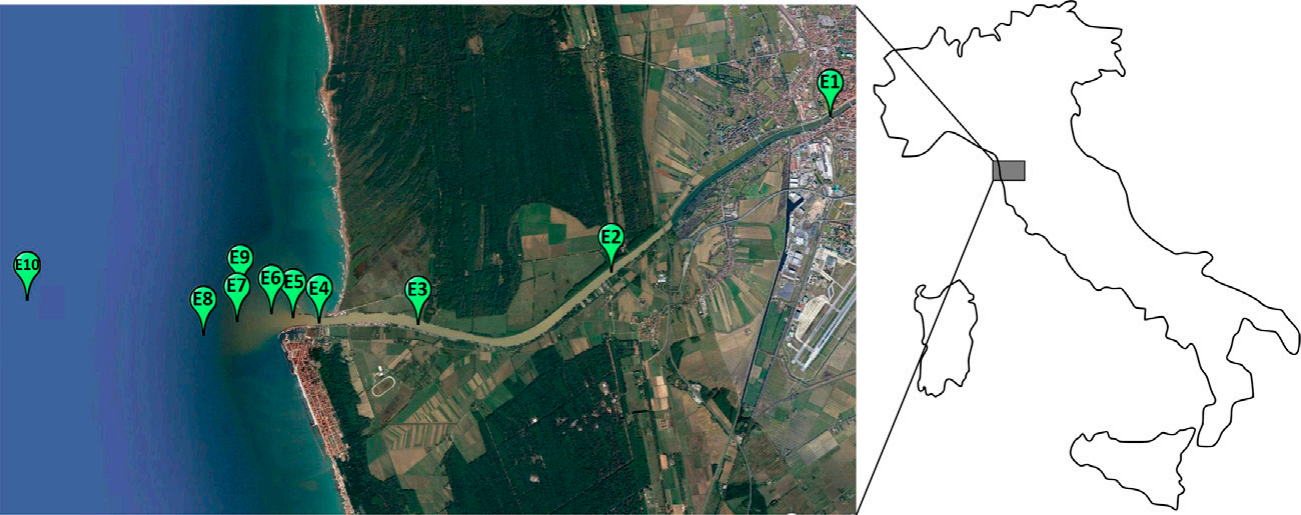
Sampling sites along the Arno River estuary (Pisa, Italy) (image source: Google Earth Pro).

For this study, the sampling was carried out at the Arno River estuary (Figure 1). The estuary region is 12 km long, highly stratified, and characterized by river flow extremes measured from 6 m^3^ s^−1^ during summer and up to 2000 m^3^ s^−1^ in winter [average discharge of 82.4 m^3^ s^−1^ (Retelletti Brogi et al., 2020)].

The lower part of the estuary hosts numerous anchored recreational/sailing boats, whose antifouling paint is a source of copper (Cindrić et al., 2015). This can potentially have unfavorable biological effects on the ecosystem in the estuary and the coastal region. Sampling was carried out in the periods of the year when the most intensive nautical traffic and the highest biological activity was expected (September 27, 2015, and April 5, 2016). Surface samples were collected at 10 sites along the salinity gradient (Figure 1).

### Samples Collection

Surface samples (depth ∼0.5 m) were collected using a van Dorn type 2.2 L horizontal water sampler (Wildco). Bottles for the sampling and sample storage for dissolved Cu measurement and Cu speciation analyses (FEP, fluorinated ethylene propylene, or PFA, perfluoroalkoxy, Nalgene) were previously cleaned with 10% HNO_3_ (analytical reagent grade), rinsed several times with ASTM Type I water (labeled hereafter as Milli-Q water, 18.2 MΩ, Millipore, USA), and finally filled with Milli-Q water until use. FEP/PFA bottles were thoroughly rinsed with the sample and 1 L of a sample was then immediately taken for on-board filtration. Samples for dissolved organic carbon (DOC), chromophoric dissolved organic matter (CDOM), and fluorescent dissolved organic matter (FDOM) analyses were collected into 2 L acid-washed polycarbonate bottles (Nalgene) and kept refrigerated and in the dark.

Vertical profiles of the main physicochemical parameters (salinity, temperature, pH, and dissolved oxygen) along the salinity gradient were measured *in situ* at each site using a Hydrolab DS5 multiparameter (CTD) probe. The CTD probe was calibrated before each campaign.

### Samples Preparation

For the determination of dissolved Cu concentrations and Cu speciation studies (determination of Cu complexing capacity, CuCC), samples were filtered on-board using precleaned (acid + Milli-Q) 0.22 μm cellulose-acetate syringe filters (Minisart, Sartorius). For the analysis of total dissolved Cu concentrations, samples were acidified in the Lab with trace metal grade nitric acid (*TraceSelect*, Fluka) to pH < 2 and within the next few days UV-irradiated (150 W medium pressure Hg lamp, Hanau, Germany) directly in the FEP/PFA bottles for at least 24 h in order to decompose natural organic matter (Omanović et al., 2006; Monticelli et al., 2010; Cindrić et al., 2015). Samples for Cu speciation studies were kept at natural pH (pH = 8.2 ± 0.1) and stored in the fridge (+4°C) until analysis which was performed within 2 months.

Samples for DOC, CDOM, and FDOM were filtered in the laboratory (within 4 h of sampling) through a 0.2 μm pore size filter (Whatman Polycap, 6,705–3,602 capsules) and dispensed into 3 × 60 ml acid-washed polycarbonate (Nalgene) bottles, used as analytical replicates. DOC, CDOM, and FDOM were immediately measured after filtration. The filtration system (syringe + both filter types) was selected after several tests with Milli-Q water, since the filtered water showed no contamination with Cu or DOC, CDOM, and FDOM.

### Determination of Dissolved Cu

Concentrations of dissolved Cu were determined by differential pulse anodic stripping voltammetry (DPASV). Measurements were carried out on Metrohm-Autolab (EcoChemie) potentiostat (PGSTAT128N) controlled by GPES 4.9 software in a three-electrode cell (663 VA Stand, Metrohm). Ag|AgCl|sat. NaCl electrode was used as the reference electrode, a Pt wire as the auxiliary, and a hanging mercury drop (HMDE) as the working electrode. The DPASV parameters used for the measurement of DCu are presented in Supplementary Table S1. Analyses were performed using a fully automated system consisting of the instrument, a home-made autosampler, and five Cavro XL 3000 syringe pumps (Tecan, United States). For the preparation of the project file for the GPES software, as well as for the treatment of the voltammograms and final calculations, the handling software was developed (https://sites.google.com/site/daromasoft/home/voltaa).

Concentrations of trace metals were determined by means of the standard addition method. A certified “Nearshore seawater reference material for trace metals,” CASS-5 (NRC CNRC), was used for the validation of the Cu analysis. The obtained value (±standard deviation) was 0.370 ± 0.030 μg L^−1^ (certified value is 0.380 ± 0.028 μg L^−1^).

### Copper Speciation Analysis: Determination of Cu Complexing Capacity (CuCC)

For the determination of CuCC, the DPASV method was used (Louis et al., 2008). The experiments were performed using the same electrochemical system as described in the previous section (excluding the use of the autosampler). The parameters used for the DPASV measurements of electrolabile Cu are presented in Supplementary Table S1. In order to avoid the adsorption of Cu into the walls of cell compartments, a quartz cell was used (Cuculić and Branica, 1996). Three automatic burette systems were used to automate Cu titration (XL 3000 syringe pumps).

The titrations of natural samples were performed at a pH of 8.2 buffered with 0.01 M borate/ammonia buffer (the final concentration in the cell). The electrochemical cell was conditioned for ∼1/2 h with 15 ml of the sample before a new 10 ml aliquot of the sample was measured. The titrations were performed by measuring the ambient Cu concentration (without Cu addition) and by increasing Cu concentration up to the maximum of 300 nM of total Cu concentration ([Cu]_T_) (using 10^−5^ M and 10^−4^ M Cu stock solutions prepared by appropriate dilutions of an atomic absorption spectrometry standard solution, TraceCERT, Fluka). Each titration was composed of a total of 15 separate points (measured in triplicate), with the Cu concentrations equally distributed in logarithmic scale, i.e., similar increments in log [Cu]_T_ (Garnier et al., 2004; Louis et al., 2009). Final points that deviated from the linear part of the titration curve were discarded, and thus some of the final titration curves were composed of less than 15 points (12–14). After each Cu addition, 30 min was estimated as enough time to reach the equilibrium conditions in the titration cell. For each Cu addition, 3 repetitive measurements were performed and peak intensities of all the points were thereafter used for the construction of complexometric curves. Before each titration, a Milli-Q test was performed to check the procedural blank. The concentration of Cu in Milli-Q was always below 0.1 nM. The theoretical background of the complexometric parameter calculation can be found elsewhere (Garnier et al., 2004; Omanović et al., 2015; Pižeta et al., 2015). Obtained complexometric curves were then treated by using the ProMCC software tool (Omanović et al., 2015). The number of ligand classes was estimated based on the shape of the Scatchard plot. For all titrations, the Scatchard plot showed a clear convex shape, indicating the presence of the 2-ligand classes. Complexation parameters were calculated by a nonlinear fitting of the Langmuir-Gerringa isotherm in a "logarithmic mode" (Omanović et al., 2015). The ambient speciation of Cu ([Cu^2+^] [CuL_1_] and [CuL_2_]) originally present in the water sample was calculated from the concentrations of total dissolved Cu ([DCu]), [L]_i_, and the conditional stability constants (log*K*_i_) of the Cu-binding organic ligands in the sample.

Model experiments with isolated organic matter (4 mg L^−1^ fulvic acid (FA), IHSS, batch 2S101F) were performed in organic-free, UV-digested seawater (UVSW) at the same pH as samples (pH = 8.2) adjusted with the borate/ammonia buffer. The UVSW was cleaned by MnO_2_ slurry following the common procedure (van den Berg, 2006).

### Dissolved Organic Carbon (DOC) and Dissolved Organic Matter Optical Properties Measurements

The DOC concentration was determined by high-temperature catalytic oxidation using a Shimadzu TOC-VCSN carbon analyzer (Santinelli et al., 2015). Prior to oxidation, samples were acidified with 2 M high purity HCl and purged for 3 min with pure air to remove inorganic carbon. To achieve satisfying analytical precision (±1%), up to 5 replicate injections were performed. The instrument performance was verified by comparison of data with the DOC Consensus Reference Material (CRM) (Hansell, 2005) (batch #13/08-13, nominal concentration: 41–44 µM measured concentration: 43.2 ± 1.5 µM, *n* = 4). A calibration curve was measured with potassium hydrogen phthalate as the organic standard.

UV-Vis absorbance spectra were measured using a Jasco UV-visible spectrophotometer (Mod-7850) equipped with a 10 cm quartz cuvette, following the method reported by (Retelletti Brogi et al., 2015). Scans were performed at excitation wavelengths of between 230 and 700 nm. The spectrum of Milli-Q water, measured in the same conditions, was used as blank and subtracted from each sample. Absorbance spectra were treated by using the ASFit tool (Omanović et al., 2019). Absorbance at 254 nm (*A*_254_) was expressed as the absorption coefficient (*a*_254_) in m^−1^ (Stedmon and Nelson, 2015). The specific UV absorbance at 254 nm (SUVA_254_) was calculated by dividing the absorption coefficient at 254 nm by the DOC concentration (m^2^ g^−1^ C) and used as an indicator of the percentage of CDOM in the total DOM pool (Marcinek et al., 2020).

Fluorescence excitation-emission matrices (EEMs) were recorded using the Aqualog spectrofluorometer (Horiba-Jobin Ivon) in a 1 × 1 cm quartz cuvette as described in detail by (Retelletti Brogi et al., 2020). Briefly, EEMs were scanned at the excitation wavelength range of 250–450 nm with 5 nm increments and emission wavelengths ranged between 212 and 619 nm with 3 nm increments. Procedural blanks were checked by measuring EEM of Milli-Q water. Fluorescence intensities were normalized to Raman units (R.U.) using daily measured Raman peak of Milli-Q water (*λ*_ex_ = 350 nm, *λ*_em_ = 371–428 nm) (Lawaetz and Stedmon, 2009). Parallel factorial analysis (PARAFAC) was applied to identify the different components in the FDOM pool by using the decomposition routines for EEMs drEEM toolbox (version 0.2.0 for MATLAB (R2016a) (Murphy et al., 2013).

## Results and Discussion

### Study of SAS Influence on the Voltammetric Signal of Cu

#### Problem Description

Initial examinations of the samples for the determination of the Cu speciation using the DPASV method with high DOC concentration (>2 mg L^−1^) revealed that a characteristic Cu oxidation peak at the ambient Cu concentration is not present (even at relatively high Cu concentrations, e.g., 20–50 nM) and that an unusually high background current is obtained. The lack of a Cu oxidation peak was rather expected because the majority of the Cu present in the samples with a high DOM load is predicted to be complexed by strong organic ligands, which are not reducible at the applied deposition potential.

**FIGURE 2:**
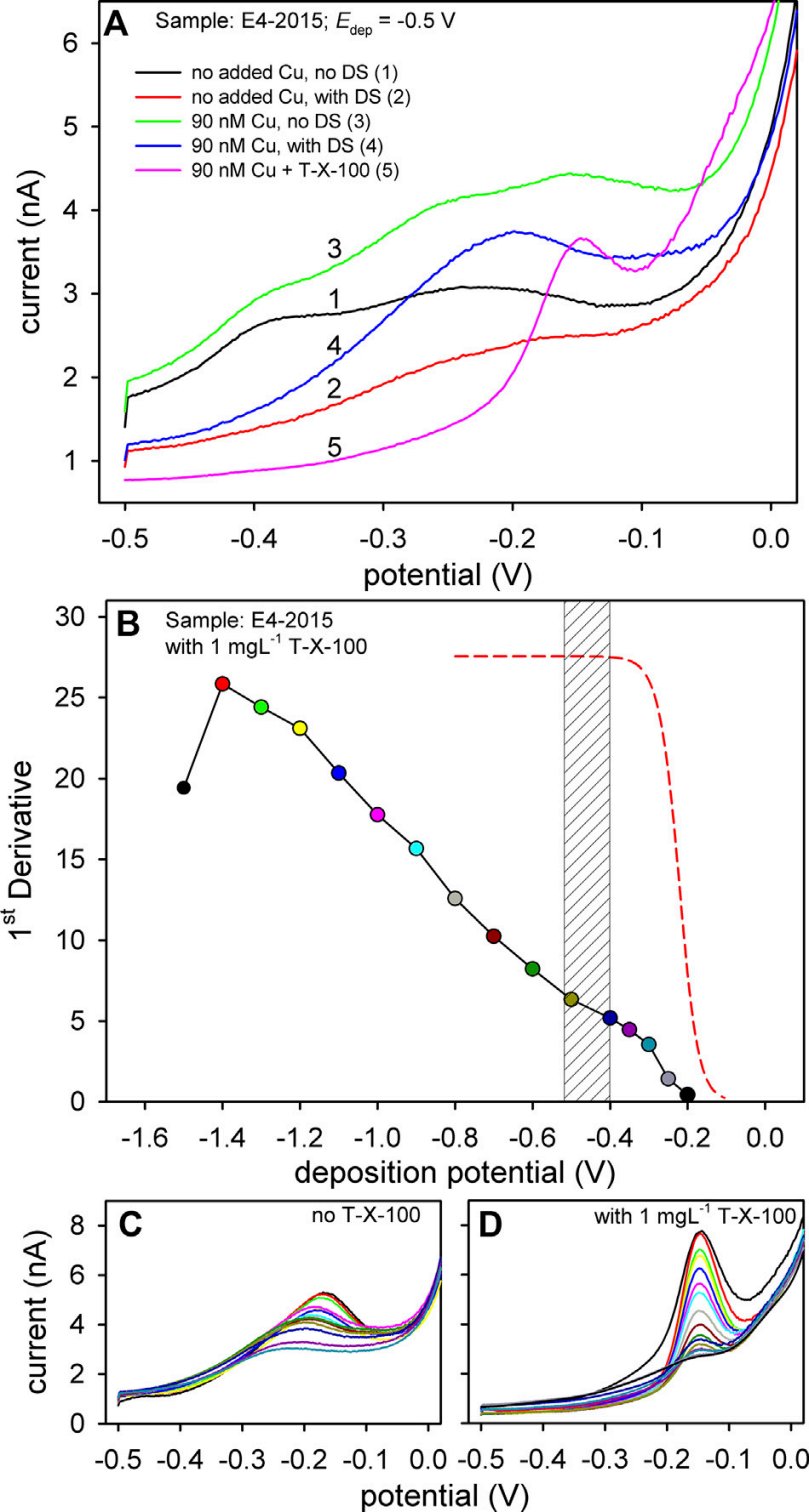
**(A)** DPASV curves obtained at various scanning conditions, solution compositions, and Cu concentrations as indicated in the figure. **(B)** Pseudopolarogram of Cu obtained in sample taken at the site E4 (2015) with [Cu]_T_ = 90 nM and with the addition of 1 mg L^−1^ T-X-100. The red dashed line illustrates the Cu pseudopolarogram without organic matter. Bottom plots represent the obtained voltammograms of pseudopolarographic measurements ([Cu]_T_ = 90 nM) without the addition of T-X-100 but using the desorption step **(C)** and with the addition of 1 mg L^−1^ T-X-100 **(D)**. Different colors represent voltammograms obtained at different deposition potentials.

However, the unusually high background current, with one or two wide peaks, obtained in most of the samples, signified a potential problem. This is clearly visible in curve #1 in Figure 2A. In this example, the voltammetric procedure consisted specifically of only the deposition step at *E*_dep_ = −0.5 V, in the sample without any addition, except the borate buffer. The high background current in voltammetric measurements as the result of adsorbed SAS is not unusual, as it was shown for several metals (Scarano and Bramanti, 1993; Louis et al., 2008; Pađan et al., 2019; Pađan et al., 2020). To overcome problems caused by SAS adsorption, a "desorption step" (DS) (the application of a very negative potential, e.g., −1.5 V, for a short period, e.g., 1–3 s), at the end of the deposition period was proposed (Louis et al., 2008). However, in our case, the application of DS did not provide the expected result due to the high content of organic matter, i.e., SAS present in the samples. Although a much better signal was obtained by applying a DS (curve #2, Figure 2A), the shape of the voltammogram at the potential range of the anodic Cu peak was still not adequate for the extraction of its intensity. The undefined wide stripping peak at the given potential was present even if the deposition of Cu was performed at potentials more positive than its redox potential; *E*_dep_ = > −0.2 V (either with or without the DS). The problem of the strange background current was even more apparent when the measurements were performed with the addition of Cu. Without the DS, a totally undistinguishable voltammogram was obtained at 90 nM Cu concentration (curve #3, Figure 2A). Although considerably better, the scan with the DS still produced a wide and poorly resolved peak (curve #4, Figure 2A). Intensities extracted from such voltammograms would be questionable, and consequently, constructed complexation curves and obtained complexation parameters would be entirely unreliable.

To resolve the problem of the adsorbed layer of the SAS on the Cu voltammetric signal, the idea was to introduce a competitive adsorptive process. The used compound should have a competitive effect on SAS adsorption but not on the Cu speciation chemistry in the bulk of the solution. Thus, a nonionic surfactant Triton-X-100 (T-X-100) was chosen for this purpose, since it does not form a complex with Cu (Krznarić et al., 1994; Omanović et al., 1996; Plavšić et al., 2009). The modified procedure employed here consists of adding T-X-100 at a concentration of 1 mg L^−1^ to the sample being analyzed. It has already been shown by several authors that T-X-100 could be beneficial in resolving some of the voltammetric issues. For example, T-X-100 was used for the voltammetric determination of iodine in seawater samples (Luther et al., 1988; Žic et al., 2012), for the separation of poorly resolved redox processes of Cu and Cu-EDTA (Omanović et al., 1996), and for the enhancement of the Cu oxidation by destabilizing formed CuCl_2_^−^ during the anodic scan (Plavšić et al., 1994). The benefit of adding T-X-100 to our samples is illustrated by curve #5 in Figure 2A. Compared to all other curves in the graph, a clearly resolved and narrow anodic Cu peak was obtained, from which extraction of Cu peak intensities is much more convenient.

For characterization of the interaction of metals with organic ligands, pseudopolarographic measurements (known also as stripping chronopotentiometry at scanned deposition potential, SSCP, or scanned stripping voltammetry, SSV) are proposed using either Hg (drop or film) or a solid electrode (Lewis et al., 1995; Luther III et al., 2001; Omanović and Branica, 2004; Town and van Leeuwen, 2004; Serrano et al., 2007; Domingos et al., 2008; Gibbon-Walsh et al., 2012; Bi et al., 2013; Town and van Leeuwen, 2019). The methodology has advanced in recent years, enabling the full characterization of the SSCP/pseudopolarographic waves (Serrano et al., 2007; Pinheiro et al., 2020) providing the heterogeneity of labile macromolecular metal-organic complexes. The full characterization of Cu complexes still has to be done using this methodology. As such, it is often used to make a "fingerprint" of the sample (Louis et al., 2008; Nicolau et al., 2008; Domingos et al., 2016). In Figure 2B, an example of the Cu pseudopolarogram obtained in an estuarine sample (E4, 2015) is presented, along with the illustrated pseudopolarogram representing the expected shape of labile inorganic Cu in the absence of organic matter (red dashed line). At this point, our goal with pseudopolarographic measurement is only to show the inherent complexity of Cu speciation by using ASV and adequately present the influence of SAS on its voltammetric behavior. The pseudopolarogram presented in Figure 2B served as an example of the sample "fingerprint" and to support the estimation of adequate deposition potential for the Cu complexation measurements (shaded region). Based on the basic features of presented waves (the shift of the pseudopolarograms toward negative potentials upon complexation with inorganic/organic ligands or its inclination) (Lewis et al., 1995; Serrano et al., 2007; Bi et al., 2013; Pinheiro et al., 2020), there is clear evidence that the reduction of various Cu-organic complexes occurs along the scanned deposition potentials. The increase of the intensity could be explained by the progressive reduction of various Cu-organic complexes (heterogeneous binding sites). Note that the presented pseudopolarogram was obtained with the addition of T-X-100. The corresponding voltammograms from which the pseudopolarogram was constructed are presented in the right bottom plot, clearly showing the fully resolved Cu peak at the same position. The construction of an analog pseudopolarogram in the sample without T-X-100 was not possible due to inadequate Cu anodic peaks, even with the applied DS step (left bottom plot). A clear difference between voltammograms with and without the added T-X-100 is shown here and is evident during Cu titrations as well (Supplementary Figure S1). It is interesting to point out the drop of the peak intensity of the point obtained at *E*_dep_ = −1.5 V. Namely, from this to more negative potentials, the adsorption of T-X-100 strongly decreases (Omanović et al., 1996; Sander and Henze, 1996), and its benefit on the shape of Cu anodic peak weakens (top black voltammogram at right bottom plot).

Although the positive effect of the T-X-100 addition was significant, we kept the desorption step as a procedural parameter because it was found that it does not have a negative effect on the final voltammogram if applied for only 1 s. However, the potential of the DS should not be more negative than −1.5 V and it should be as short as possible to avoid complete desorption of T-X-100.

To verify the proposed method, additional test experiments were performed in "organic-free" seawater (UVSW) with and without added fulvic acid as a model of natural organic matter.

### Verification of the Methodology

#### Organic-Free Seawater

As noted previously, it is very important that added T-X-100 does not have a competitive effect on Cu complexation by natural ligands (especially organic). If that was the case, the effect would be visible on titration curves and pseudopolarograms. According to the basic model experiments, presented in the following sections, this is not the case.

**FIGURE 3:**
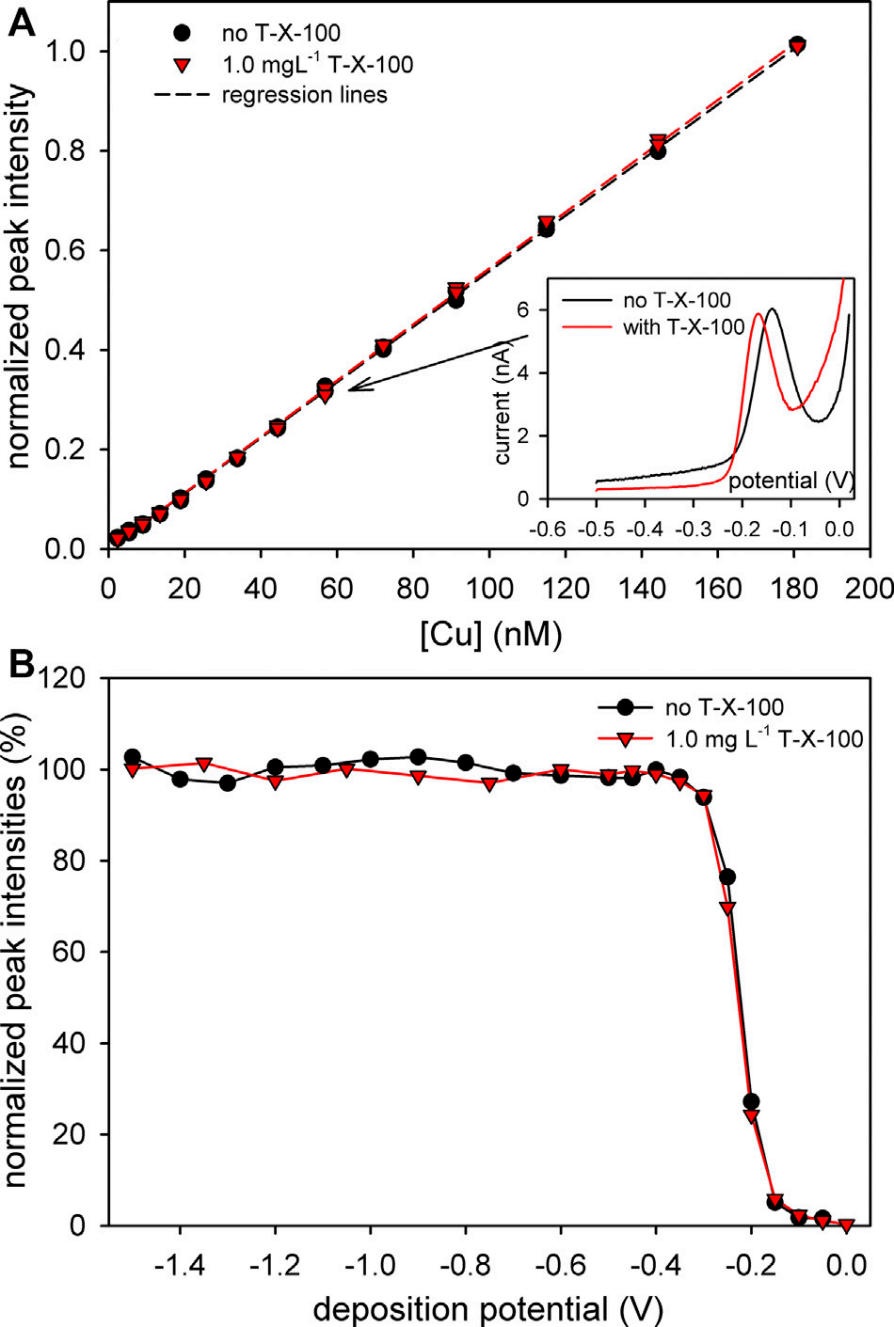
**(A)** Copper titration curves obtained in UVSW (pH = 8.2) with and without the addition of 1 mg L^−1^ T-X-100 (for better comparison, the peak intensities were normalized on the highest value). Inset: Cu voltammograms with and without T-X-100 corresponding to Cu concentration indicated by the arrow. **(B)** Normalized pseudopolarograms obtained with and without the addition of 1 mg L^−1^ of T-X-100 (intensities normalized to the estimated limiting value).

Titrations with increasing Cu concentrations were first performed in UVSW (i.e., in the absence of organic ligands) with and without added T-X-100. As shown in Figure 3A, normalized intensities (with and without added T-X-100) showed the same linear relationship with increasing concentration of Cu. Normalized intensities (the 1st derivative as an analytical signal) were used for better comparison. In both cases, well-resolved Cu anodic voltammograms were obtained (inset in Figure 3A) and there is no curvature shape at the foot of the titration curves, characteristic for titration of samples with organic ligands (Pižeta et al., 2015), indicating that there is no complexation of Cu by T-X-100. The small negative shift and a slightly narrower anodic peak obtained with T-X-100 could be explained by the destabilization of formed CuCl^2-^ during the anodic scan (Plavšić et al., 1994).

Furthermore, in cases where Cu forms a complex with T-X-100, a shift and/or a change in the shape of pseudopolarograms at a potential range more negative than the reduction potential of inorganic Cu would be expected (Lewis et al., 1995; Omanović, 2006; Gibbon-Walsh et al., 2012; Pinheiro et al., 2020). However, the pseudopolarograms obtained with and without the addition of T-X-100 showed almost the same shape (Figure 3B). This is in slight contrast from the experiment performed by (Sahlin and Jagner, 1996) using striping potentiometry with a mercury film electrode with a fully saturated T-X-100 layer (1,000 mg L^−1^) in which a slight shift of the pseudopolarographic wave was observed. However, the concentration of T-X-100 used in their experiment was 1,000× higher (1 g L^−1^) than in our work and as such is not directly comparable. Therefore, based on our experiments presented in Figure 3, it can be assumed, with great confidence, that, at the concentration used in our work, T-X-100 did not form a complex with Cu and as such it works as "interference-free" for Cu speciation studies using ASV.

#### FA as a Model of Natural Organic Matter

**FIGURE 4:**
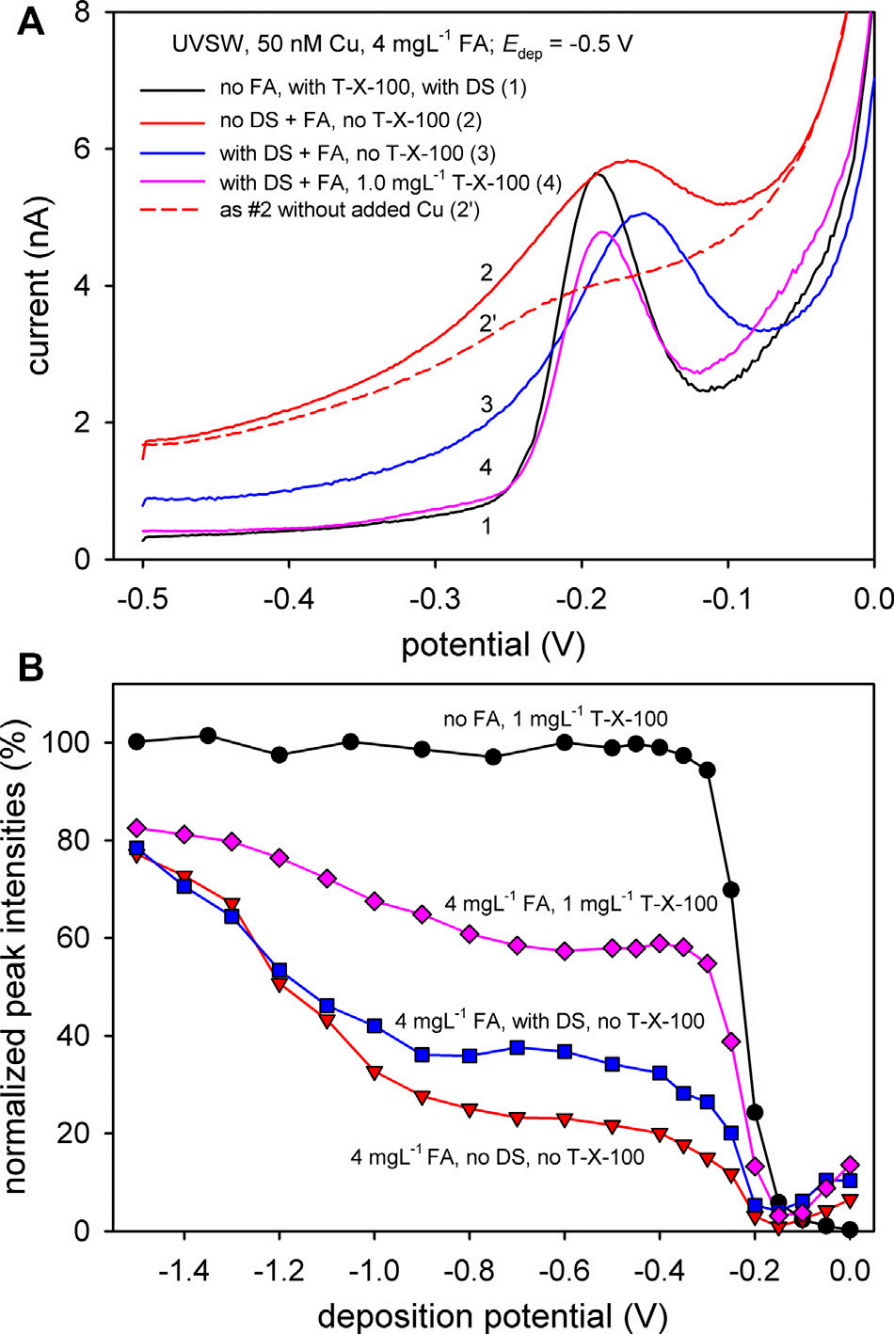
**(A)** DPASV curves obtained in UVSW at various scanning conditions and solution composition as indicated in figure. **(B)** Pseudopolarogram of Cu obtained in UVSW at various scanning conditions and solution composition as indicated in the figure.

Further examination of the proposed methodology using T-X-100 (1 mg L^−1^) was performed in UVSW with the addition of 4 mg L^−1^ of FA as a model of terrestrial organic matter showing considerable surface activity and 50 nM of added Cu (all experiments performed at pH = 8.2). The selected voltammograms are presented in Figure 4A and corresponding pseudopolarograms in Figure 4B. For comparison purposes, a voltammogram without FA (but with T-X-100) is presented by curve #1 in Figure 4A. In the sample with 4 mg L^−1^ FA and 50 nM of Cu and without the applied DS, a relatively high background current was obtained, with a relatively wide Cu peak (curve #2, Figure 4A).

Compared to the poorly shaped Cu signal in the real estuarine sample presented in Figure 2A, this peak seems easily resolvable despite the curvature baseline under the peak. However, the voltammogram in the presence of FA without added Cu shows a small peak at a slightly more positive potential (curve #2′, Figure 4A), making the manual construction of the curvature baseline more complicated. The above-mentioned problem in the Cu intensity determination is more profound at the low Cu intensities, which correspond to the foot of the titration curve where strong complexing ligands dominate. This in turn may introduce a high uncertainty in the determination of the complexation parameters, making them ultimately unreliable (Omanović et al., 2010). By applying DS, a better resolved and higher Cu peak is obtained (curve #3, Figure 4A). It was shown that the main influence of the adsorbed FA occurs during the stripping step, with Cu-FA considered as a labile complex (Town, 1997). Due to the nature of the differential pulse technique, Cu oxidized during the applied pulse is being progressively concentrated in the vicinity of the electrode surface and immediately complexed by the accumulated organic ligands, causing the shift of the oxidation peak to a more negative potential (Town, 1998). This scenario is likely to occur taking into account the fact that the Cu complex with FA is directly reducible (Whitby and van den Berg, 2015). The lability of Cu-FA complexes and problems associated with DPASV measurement of Cu in the case of adsorbable ligands, such as FA, are already documented in the literature (Soares and Vasconcelos, 1994; 1995).

The Cu stripping peak was further improved by the addition of T-X-100 (curve #4, Figure 4A). The same shape and the peak potential in presence of FA, after the addition of T-X-100, as the one without added FA (curve #1, Figure 4A) indicate that T-X-100 practically eliminated the influence of the SAS adsorbed during the stripping step.

The problems associated with the stripping voltammetry of Cu in the presence of a high concentration of organic matter are reflected in the shape of the pseudopolarograms. As presented in Figure 4B, a clear reversible pseudopolarographic wave of Cu was obtained in the absence of FA. Upon the addition of FA, the form of the pseudopolarogram considerably changed. For all pseudopolarograms with added FA, the decrease of the intensity of the initial value without FA was around 20% at a high deposition potential (*E*_dep_ = −1.5 V). However, in the potential range between −0.8 and −0.4 V, the decrease of the intensity was much higher: ∼ 40% in the presence of 1 mg L^−1^ T-X-100 and even ∼75% (w/o DS) and ∼65% (with DS) in the absence of T-X-100. The decrease of intensities at more positive potentials is already reported in the literature (Town and Filella, 2000; Gibbon-Walsh et al., 2012), indicating the complexation of the Cu with the added FA.

As mentioned earlier, the shift of the pseudopolarographic wave in the case of labile reversible Cu complexes, or those which are irreversibly reduced, could be used to determine the stability constants of formed complexes by using the so-called "chelate scale" (Lewis et al., 1995; Omanović, 2006; Gibbon-Walsh et al., 2012). With this in mind, the steep increase of intensities in pseudopolarograms without T-X-100 at potentials more negative than −0.9 V may indicate the presence of very strong organic complexes with high stability constants which are irreversibly reduced (Lewis et al., 1995; Branica and Lovrić, 1997; Gibbon-Walsh et al., 2012). This increase was not observed in experiments with Cu-FA performed using a gold-vibrating electrode (Gibbon-Walsh et al., 2012), whereas in experiments of (Town and Filella, 2000) or (Chakraborty et al., 2007), this range of deposition potentials was not scanned and could not be compared. If the metal-to-ligand ratio in the bulk of the solution used in the work of (Gibbon-Walsh et al., 2012) is compared to our ratio, the extent of the decrease of intensities at *E*_dep_ range between −0.8 and −0.4 V in our experiment is larger than expected. This is probably caused by the above-discussed strong influence of accumulated FA on the stripping process, which is much stronger on a Hg electrode than on the vibrating Au-microelectrode. The observed increase of intensities toward more negative potentials could also be partially related to the decreasing FA adsorption. This is supported by the progressive shift of Cu peak potentials (up to ∼ Δ20 mV between −1.5 and −0.7 V) in voltammograms used for the construction of pseudopolarograms without DS (data not shown). With the applied DS, the shift was only ∼ Δ5 mV, which confirms that DS removes the accumulated FA to a great extent. There was "only" ∼ 40% decrease of intensity (of the initial value without FA) at *E*_dep_ range between −0.8 and −0.4 V after the addition of T-X-100 (with no change in the Cu peak potentials across the scanned range), which is much closer to the values obtained by Gibbon-Walsh et al. (2012). Even after the addition of T-X-100, the small increase of intensities, from *E*_dep_ = −0.9 V to more negative values, remained. This is also evident in the pseudopolarogram of the natural estuarine sample (Fig. 2B). It indicates that 1) there is still a portion of FA adsorbed during the deposition step or 2) a portion of the strong Cu-FA complexes are irreversibly reduced at this range of potentials (Lewis et al., 1995; Gibbon-Walsh et al., 2012). The detailed examination of Cu-FA interactions is beyond the scope of this paper and was not further studied.

### Copper Speciation in the Estuarine Samples

**FIGURE 5:**
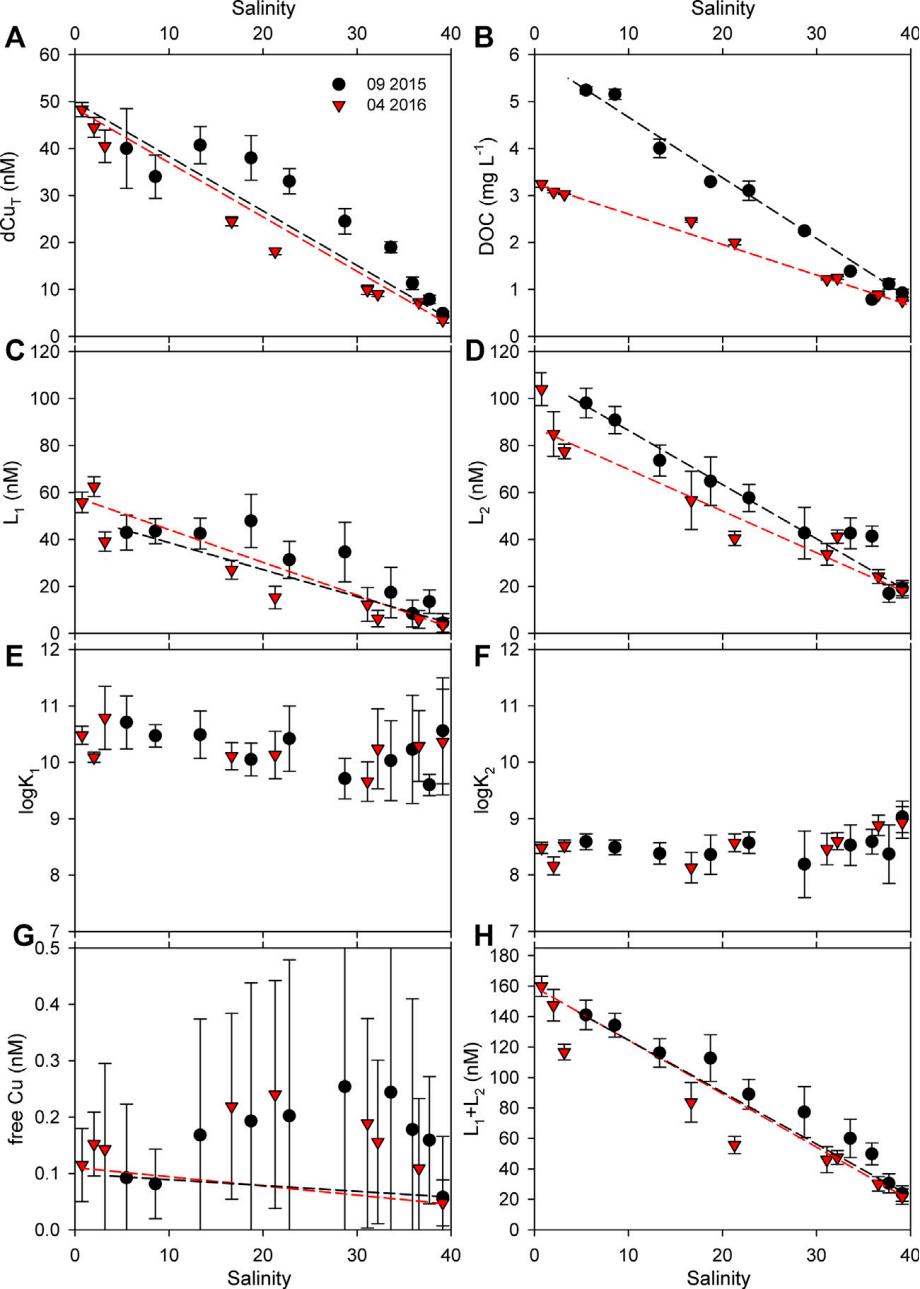
Measured parameters along the estuarine segment for two sampling periods. Uncertainties are expressed as 95% confidence intervals. Dashed lines represent the projected conservative trends.

Figure 5 presents all the results relevant for the Cu speciation study in samples collected for two different periods, the early spring and the late summer.

#### Total Dissolved Copper

A strong decrease of dissolved copper (DCu) concentrations with increasing salinity was observed for both campaigns with a slightly different distribution in the salinity gradient (Figure 5A). For the late summer campaign (September 2015), which is characterized by a lower river discharge (as evident from the salinity at the first site, E1, and the salinity contour plot in Supplementary Figure S2) (Retelletti Brogi et al., 2020), there is an increase of Cu concentration at the site E3, most likely caused by an anthropogenic input of Cu. Namely, numerous boat anchorage sites are found in the part of the estuary that begins between sites E2 and E3 (Supplementary Figure S3). In the summer period, these locations were fully occupied by boats. Thus, the observed increase of Cu in that region could be ascribed to the leaching of the Cu from the boats' antifouling paints, in which Cu is used as the biocide component (Turner, 2010; Cindrić et al., 2015). In the spring campaign (April 2016), due to the absence of the anchored boats (Supplementary Figure S3), Cu input was low. In this period, removal of dissolved Cu along the salinity gradient is implied by its deviation from the conservative line. This is probably the result of the flocculation process, which is common at lower salinities (Sholkovitz, 1976; Waeles et al., 2008). The concentrations of dissolved Cu in the seawater end-member for both periods reached the range characteristic for a clean coastal and open Mediterranean Sea area (Oursel et al., 2013; Migon et al., 2020).

#### Dissolved Organic Carbon

DOC concentrations show clear conservative behavior along the salinity gradient in both campaigns, with decreasing concentrations toward the sea end-member. Removal and/or production of DOM have a small impact on the DOC concentration within the estuary, suggesting that the dilution of DOC in riverine water with marine water is the main process affecting DOC dynamics in the estuary. The DOC concentration is almost 2× higher in the late summer than in the spring campaign in the river end-member (5.2 mg L^−1^ in September 2015 in contrast to 3.3 mg L^−1^ in April 2016), whereas the sea end-member shows the same DOC values in both periods (∼0.8 mg L^−1^). In both periods, in the river end-member (at the E1 site), the measured DOC concentrations are in agreement with the DOC seasonal cycle observed in the river (Retelletti Brogi et al., 2020). High concentrations of DOC in the Arno River are the result of the weathering processes, especially during flood events, which primarily bring the terrestrial DOM, as well as the autochthonous production during spring and summer periods (Retelletti Brogi et al., 2020). Anthropogenic input of DOM also plays a role, because of the numerous anthropogenic activities in the upstream riverine region.

#### Copper-Binding Ligands and Estimation of Copper Bioavailability

Applying the adapted methodology for the determination of Cu speciation, well-shaped complexometric titration curves were obtained (examples are given in Supplementary Figure S4). Two classes of ligands were identified in all samples, denoted as L_1_ and L_2_, corresponding to a stronger and a weaker class of organic ligands, respectively. Concentrations of L_1_ ranged from 3.5 ± 2.9 to 63 ± 4 nM eq Cu, whereas L_2_ concentrations were higher and ranged from 17 ± 4 to 104 ± 7 nM eq Cu (Figures 5C,D). The concentration of both ligand classes decreased with the salinity, as was similarly observed for DCu and DOC. Along the estuary, the concentrations of ligands exceeded the corresponding total dissolved Cu concentrations, which are usually obtained in coastal regions (Bruland et al., 2000; Hurst and Bruland, 2005; Louis et al., 2009; Plavšić et al., 2009; Wong et al., 2018). The apparent stability constants (log*K*_Cu_) of the two ligand classes ranged from 9.6 ± 0.2 to 10.8 ± 0.6 for L_1_ and from 8.2 ± 0.3 to 9.0 ± 0.3 for L_2_, without a noticeable difference between the two sampling periods (Figures 5E,F). The ranges of estimated constants are typical for estuarine and coastal regions obtained by the ASV method and reflect the so-called "detection window" (DW) of the method (Apte et al., 1988; Bruland et al., 2000; Pižeta et al., 2015).

One of the purposes of the determination of metal complexing parameters is to estimate the free metal concentration, which is considered to be the most bioavailable/toxic. Having in mind the potential overestimation of free Cu using the ASV method, as will be discussed later, we refer to it as "free" Cu (quoted). In our study, the estimated "free" Cu concentrations ranged from 50 to 250 pM, following the strong positive deviation from the projected conservative line. The obtained trends are the result of the estimated complexation parameters, along with the dissolved Cu concentration. These are fairly high values of "free" Cu, taking into account the fact that the estimated threshold toxicity level is around 10 pM (Sunda et al., 1987; Gledhill et al., 1997), but as already mentioned they are likely overestimated due to the methodological problems.

The measured concentrations of the stronger ligand class (L_1_) are similar to dissolved Cu concentrations. Although in some cases L1 was almost saturated with Cu at its ambient concentration, Cu was still mainly controlled by the abundance of strong ligand class (L_1_) with high stability constants: between ∼55 and 90% of dissolved Cu exists solely in the form of strong complexes.

#### Optical Properties of DOM

PARAFAC analysis applied to all the samples revealed 5 FDOM components. The components spectra were compared with previous studies by using the Openfluor database (Murphy et al., 2014) and identified as microbial humic-like (C1), terrestrial fulvic-like (C2), protein-like (C3), terrestrial humic-like (C4), and protein + PAH-like (polycyclic aromatic hydrocarbons) (C5). In both periods examined here, the protein-like component dominates the FDOM pool (Supplementary Table S2). This is in agreement with the two-year study performed in the Arno River (Retelletti Brogi et al., 2020). The average percentage of microbial humic-like (C1) and protein-like (C3) components is approximately the same in the two seasons (25.1% and 25.1% for C1 and 31.7% and 34.0% for C3 in spring and late summer, respectively), whereas terrestrial components (C2 and C4) represent a higher average percentage of the FDOM pool in spring (21.0% for C2 and 15.8% for C4) than in late summer (14.6% for C2 and 9.6% for C4) (Supplementary Table S2), due to the higher river discharge in spring. However, the highest difference is observed for the protein + PAH-like component (C5), whose average percentage in the whole FDOM pool was 6.4% in spring and 16.7% in late summer. This can be the consequence of intense touristic activity in late summer, i.e., extensive automobile and boat traffic in the area, introducing PAH-like DOM from the exhaust. SUVA_254_ supports a change in DOM pool between the two seasons, suggesting a higher percentage of chromophoric DOM in late summer than in spring (Supplementary Figure S5).

#### Linking Copper Organic Ligands with DOM Properties

The stability constants obtained in this study appear to be lower at midsalinity range than at the end-members for both ligand types (Figures 5E,F). However, considering the associated uncertainties, there is no clear statistical difference among them. Despite that, the obtained trends could indicate the change in the composition of the organic ligands at different salinities. A change in DOM composition in the salinity gradient is suggested by the decrease of SUVA_254_ and DOC normalized PARAFAC components toward the sea end-member (excluding the C5/DOC in spring) (Supplementary Figure S5), signifying the decrease of the chromophoric and fluorescent fraction in the DOM pool (the addition of nonchromophoric DOM and/or removal of chromophoric DOM). While the sum of the ligands (ΣL) correlates with the distribution of DOC, *a*_254_, and all PARAFAC components (*r* ^2^ > 0.9), the obtained trends of stability constants in the salinity gradient do not show a strong link with any of the optical properties of DOM (CDOM/FDOM) (data not shown). Higher stability constants at a high salinity could be a consequence of the addition of organic ligands with stronger binding constants (Whitby et al., 2017) not visible by UV/Vis and fluorescent measurements. Another possibility is that removal of ASV-labile organic ligands occurs at a high salinity which could lead to a slight increase of conditional stability constants.

**FIGURE 6:**
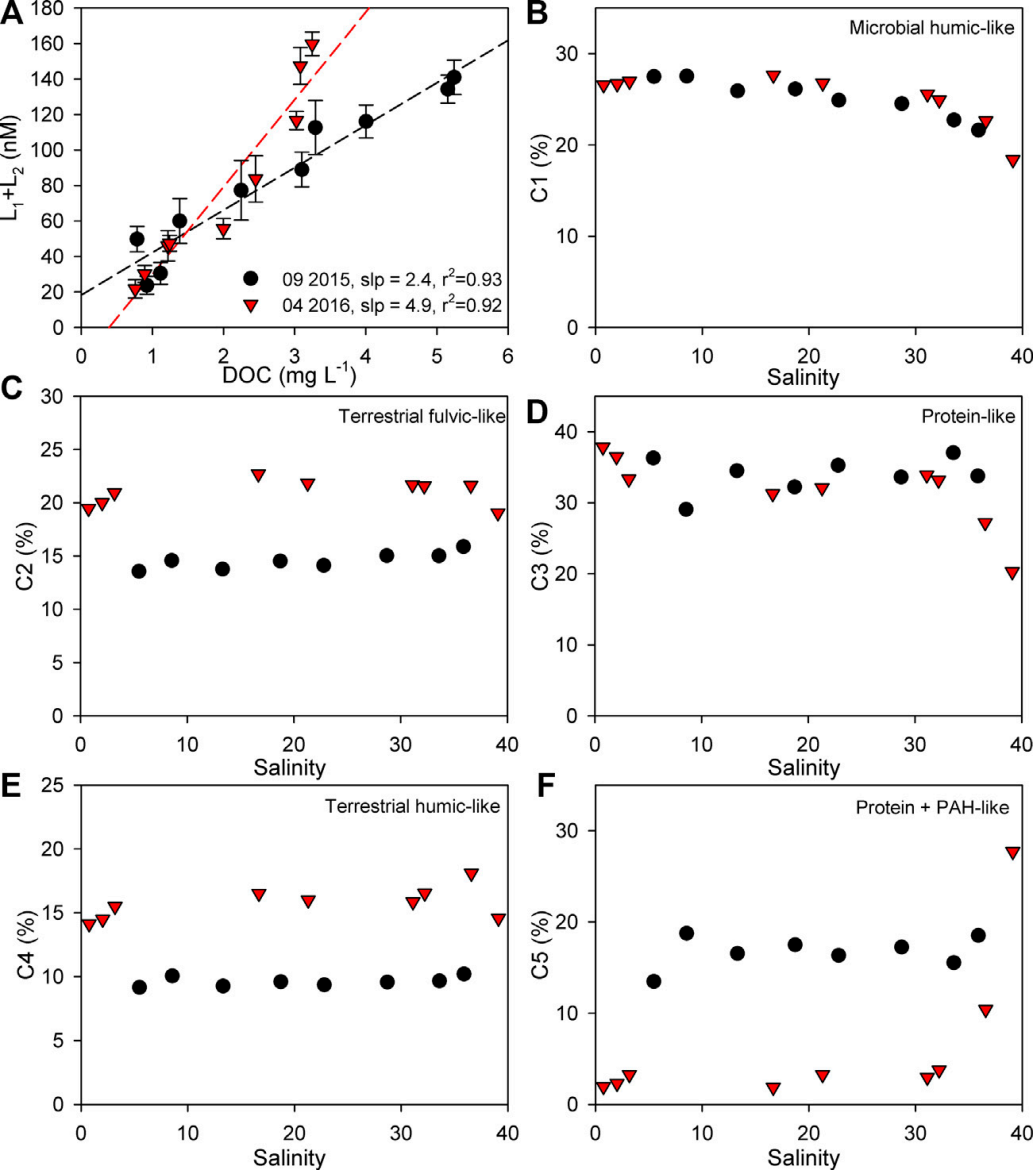
Relationship between the sum of organic ligands (L_1_+L_2_) and DOC **(A)** and the variation in the percentage of PARAFAC components (C1–C5) along the salinity gradient **(B–F)**. Dashed lines represent the linear regression of the data.

A different relationship between the sum of the ligands (ΣL) and the DOC is observed in spring and summer: the estimated slope of their relationship is ∼2× higher during spring than during summer (Figure 6A). Although the DOC, *a*_254_, SUVA_254_ and DOC normalized fluorescence intensities of all PARAFAC components (Supplementary Figure S5) are higher in late summer than in spring, the DOM pool in spring is more abundant with Cu-binding ligands. This can be linked to a higher percentage of terrestrial fulvic and humic components (C2 and C4, respectively) in the FDOM pool in spring (Figures 6C,E).

This is in agreement with other studies performed in estuarine regions, which suggest that humic and fulvic acids are the major source of organic ligands in these environments (Kogut and Voelker, 2001; Yang and van den Berg, 2009; Whitby and van den Berg, 2015; Wong et al., 2018; Dulaquais et al., 2020). Along with the humic substances, it was found that biogenic thiourea-type thiols are abundant ligands in estuarine environments (Whitby et al., 2017). A strong positive correlation between ΣL and the dissolved Cu concentration is observed in our study, which is on average the same for both periods (Supplementary Figure S6), suggesting that Cu-binding ligands could be the driver of DCu estuarine geochemistry. The increase of the organic ligands in the midsalinity range (S = 15–30), in late summer, could be a biological response to Cu release in the boat anchorage area (Zitoun et al., 2019). *In situ* production mainly releases protein-like substances (Osburn et al., 2012) and in this study, the protein component does indeed show an increase in the midsalinity range in late summer, while in the spring this is not the case. However, a similar increase is observed for all other components, showing a positive correlation with ΣL. Even though *in situ* production can also cause the increase of humic-like components (Romera-Castillo et al., 2010; Marcinek et al., 2020), without additional biological information we cannot conclude that the correlation between ΣL and the DCu in late summer is related to any extent to a biological response to Cu-stress.

#### Evaluation of Derived Complexation Parameters and Anodic Stripping Voltammetry Method Limitations

The method of calculation of complexation parameters (free and/or inorganic Cu, ligand concentrations, and conditional stability constants) in natural samples using both ASV and CLE-AdCSV provides results which are "operationally defined" (Bruland et al., 2000). Namely, the derived apparent stability constants depend strongly on the applied DW, which is expressed as the "side reaction coefficient" (*α*) (Bruland et al., 2000; Buck et al., 2012; Pižeta et al., 2015). For ASV, *α* is much smaller than it is for the CLE-AdCSV method in which the ligand with known stability constants is used as a competing ligand. In our measurements, the calculated logα is 1.13 (the ratio of the sum of all inorganic Cu species vs. free Cu concentration), whereas for CLE-AdCSV, logα depends on the used competitive ligand type and its concentration, and for Cu, it is usually logα >3 (Bruland et al., 2000; Buck et al., 2012; Whitby et al., 2017; Wong et al., 2018). Thus, if compared to CLE-AdCSV, it might seem like the stronger class of ligands are underestimated (Dulaquais et al., 2020); however, this is the consequence of DW which is not comparable to these two methods. It should also be noted that theoretically the method for calculation of complexation parameters using ASV assumes that the measured signal corresponds purely to the inorganic Cu. However, it is known that, during the deposition time at the appropriate deposition potential, kinetically labile complexes are actually accumulated, which include not only fully labile inorganic Cu species but also a portion of the kinetically labile organic complexes (Town, 1997; Bruland et al., 2000; Town and van Leeuwen, 2004; Chakraborty et al., 2007; Gibbon-Walsh et al., 2012). To prevent the reduction of labile organic complexes as much as possible, the deposition potential should be selected at the most positive value at the range of limiting currents (top of the wave) of the Cu pseudopolarogram without the addition of organic ligands (shaded area in Figure 2B). It is generally assumed that stronger complexes are less kinetically labile than the weaker complexes, and as such, it is expected that this problem will more greatly impact the weaker class of ligands.

This problem is partly reflected in the estimated "free/bioavailable" Cu. However, as Cu exists mainly in the form of strong complexes at ambient concentrations, it could be considered that the contribution of kinetically labile complexes to the bioavailable fraction is not high. Despite these potential methodological problems in the estimation of free Cu (which is the basis for the assessment of Cu toxicity), it should be mentioned that some model toxicological experiments showed a very good agreement with the labile Cu estimated by ASV and its bioavailability/toxicity (Tubbing et al., 1994; Lage et al., 1996; Tait et al., 2016; Sánchez-Marín, 2020). Furthermore, the free Cu concentrations obtained by the ASV method in estuarine samples (Jones and Bolam, 2007; Louis et al., 2009) were found to correlate well with those predicted by WHAM VII modeling (Stockdale et al., 2015), highlighting a particular feature of the ASV method.

Overall, it should be noted that each of the currently most-utilized techniques for the estimation of metal speciation (and their bioavailability) in natural waters (diffusive gradients in thin films (DGT), CLE-AdCSV, and ASV) provide results that are essentially "operationally defined"; i.e., they are technique-dependent.

## Conclusions

Here, we present an adapted ASV methodology of copper speciation in estuarine samples with high DOM concentration. The method relies on the addition of the nonionic surfactant T-X-100, which competitively inhibits the adsorption of DOM on the Hg electrode during the deposition and stripping steps. Tests performed with and without the addition of fulvic acid (FA) as a model organic ligand revealed that, at the concentration level used here (1 mg L^−1^), T-X-100 does not have any detectable influence on the redox process of Cu, labeling it "interference-free" for Cu speciation studies.

The proposed method improvement enabled the Cu speciation analysis in the Arno River estuary (Italy) characterized by high concentrations of both Cu and DOC. Mixing with the clean seawater leads to a decrease in their concentrations, reaching the value characteristic for a clean coastal region. Speciation analyses revealed the existence of the two types of organic ligands responsible for Cu complexation: a strong ligand class (L_1_) with a concentration level similar to that of dissolved Cu and a weaker one (L_2_) found at higher concentrations. The calculated free Cu concentrations in analyzed samples were above the toxicity threshold level. However, due to the ASV methodological specificities, these values might have been slightly overestimated. The sum of the ligand concentrations (ΣL) had the same linear relationship with dissolved Cu for both sampling periods. By contrast, a different relationship between ΣL and DOC was found in the two sampling periods, implying a change in the contribution of Cu-binding organic ligands to the DOM pool between seasons. The results indicate that the DOM pool comprised a higher percentage of Cu-binding ligands in spring than in summer, due to the higher percentage of terrestrial fulvic and humic components in the FDOM pool. However, as a separate quantification of specific ligand types was not performed, a direct link with detected the Cu-binding ligand classes was not possible to confirm. Assigning the exact type of the organic ligands able to complex Cu in the Arno River estuary demands more detailed studies with additional ligand characterization methods.

## Data Availability Statement

The raw data supporting the conclusions of this article will be made available by the authors, without undue reservation.

## Author Contribution

JP, CS, SB, OR, CG, and DO performed sampling and sample preparation. JP performed analyses of samples, analyzed the data, and drafted the manuscript. SM conceptualized the manuscript, organized data processing and interpretation, and wrote the manuscript. A-MC performed analyses and validation of the technique. CS and SB performed analyses of DOM and interpreted the DOM results. OR, CG, CS, and DO organized and conceptualized the study in the Arno River estuary. OR and CG acquired the funding. DO designed the experiments, supervised the method development, and acquired the funding. All authors contributed to results interpretation and manuscript writing/editing.

## Funding

This study was realized within the framework of the COMECOM project as a part of the “MerMex-WP3-C3A” project (PIs: OR and CG), as well as within the scope of the project “New methodological approach to biogeochemical studies of trace metal speciation in coastal aquatic ecosystems” (MEBTRACE), financially supported by the Croatian Science Foundation under the project number IP-2014-09-7530 (PI DO).

## Conflict of Interest

The authors declare that the research was conducted in the absence of any commercial or financial relationships that could be construed as a potential conflict of interest.
