# Supplementary material for: Organic Copper Speciation by Anodic Stripping Voltammetry in Estuarine Waters With High Dissolved Organic Matter
Source: Front Chem. 2021 Feb 2;8:628749. doi: 10.3389/fchem.2020.628749 (PMC7901884; doi:10.3389/fchem.2020.628749)
Supplement: Supplementary file 1 [file datasheet1.pdf]

Supporting information document for the manuscript:

Organic copper speciation by anodic stripping voltammetry (ASV) in estuarine waters with high dissolved organic matter

Jasmin Pađan[1], Saša Marcinek[1,*], Ana-Marija Cindrić[1], Chiara Santinelli[2], Simona Retelletti Brogi[2], Olivier Radakovitch[3,4], Cédric Garnier[5], Dario Omanović[1,*]

[1]Ruđer Bošković Institute, Center for Marine and Environmental Research, Zagreb, Croatia

[2]CNR - Biophysics Institute, Pisa, Italy

[3]Aix-Marseille University, CNRS, IRD, INRAE, Coll France, CEREGE, Aix-en-Provence, France

[4]IRSN (Institut de Radioprotection et de Sûreté Nucléaire), PSE-ENV/SRTE/LRTA, 13115 Saint-Paul-Les-Durance, France

[5]Mediterranean Institute of Oceanology, ECEM, Toulon University, La Garde, France

***Corresponding authors**: omanovic@irb.hr and smarcin@irb.hr

Table S1. Parameters for differential pulse anodic stripping voltammetry (DPASV) used for determination of total dissolved Cu concentration (DCu) and Cu speciation (CuCC) measurement.

Parameter	CuCC	DCu
Deposition potential (V)	-0.50	-0.85
Duration (s)	60-180	60-300
Desorption potential (V)	-1.5	-
Duration (s)	1-2	-
Equilibration time (s)	5	5
Modulation time (s)	0.05	0.05
Interval time (s)	0.1	0.1
Initial potential (V)	-0.5	-0.5
End potential (V)	0.0	0.0
Step potential (V)	0.002	0.002
Modulation amplitude (V)	0.040	0.040

Table S2. Average fluorescence intensity of PARAFAC components (R.U.) and their average contribution (%) to the whole FDOM pool in September 2015 and April 2016.

		**C1**	**C2**	**C3**	**C4**	**C5**
**09/2015**	R.U.	0.41	0.22	0.53	0.15	0.26
	%	25.1	14.6	34.0	9.6	16.7
**04/2016**	R.U.	0.15	0.12	0.19	0.08	0.02
	%	25.1	21.0	31.7	15.8	6.4

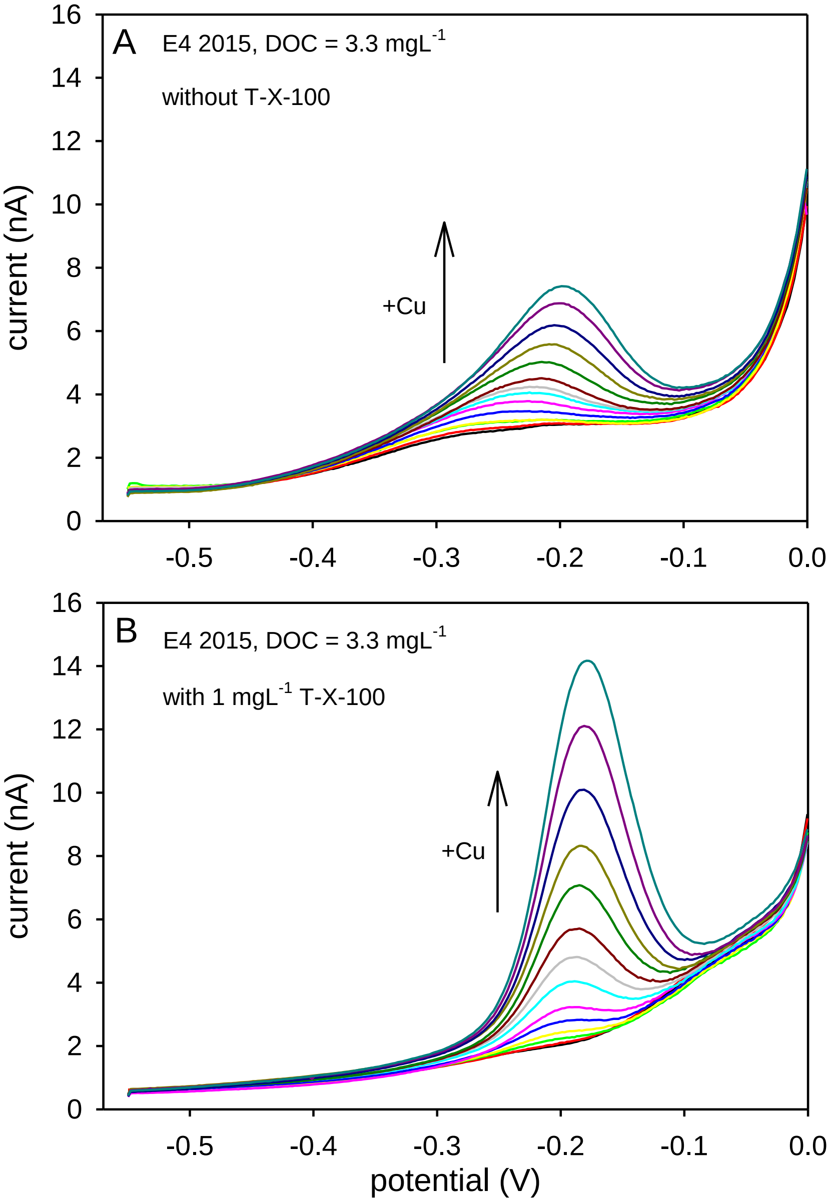

Figure S1. DPASV titration curves obtained by increasing additions of Cu in E4 (2015) sample using (A) desorption step (@E_{DS} = -1.5 V) and without added T-X-100 and (B) with addition of 1 mgL^{-1} T-X-100.

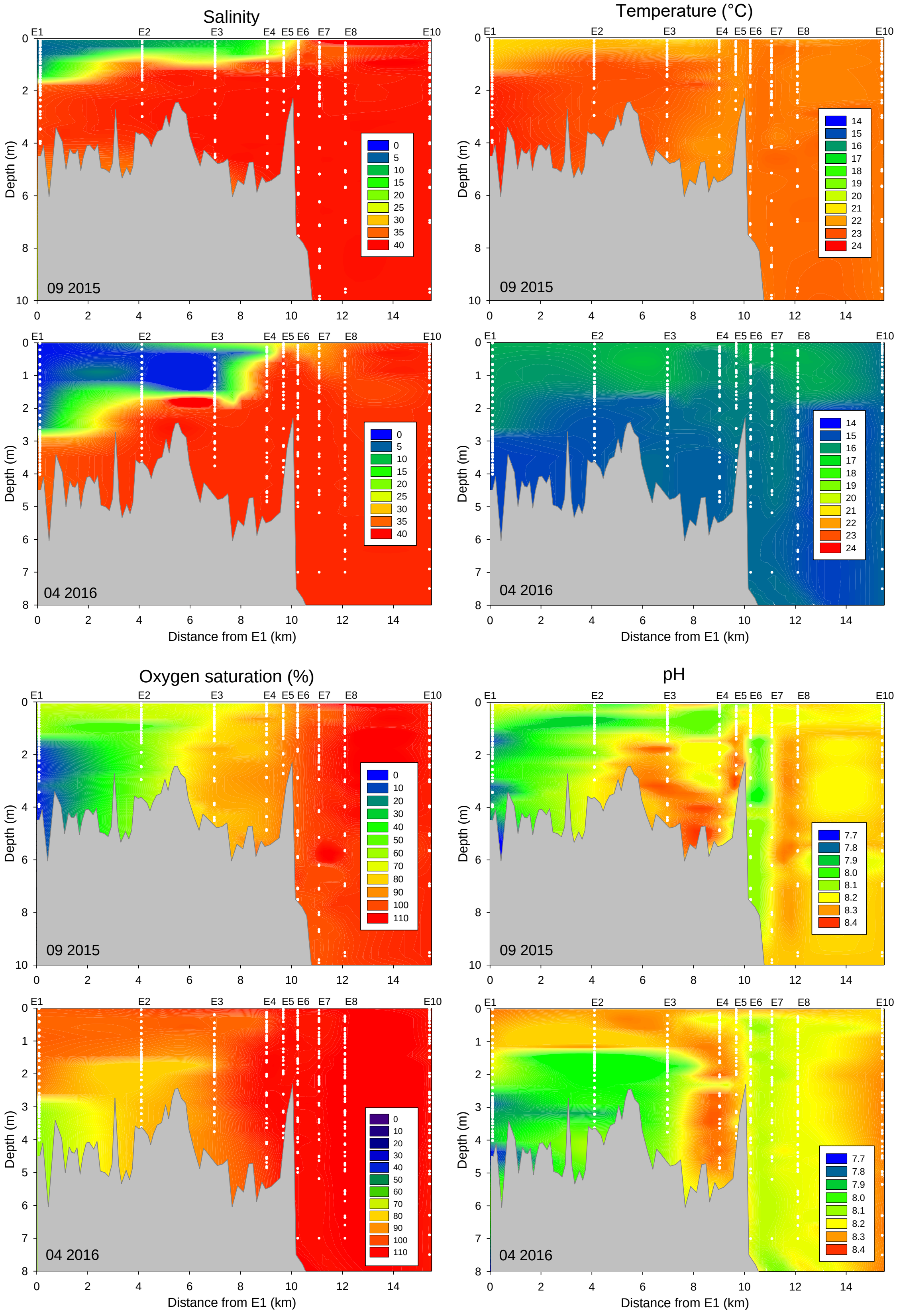

Figure S2. Contour plots of salinity, temperature, dissolved oxygen and pH along the vertical profiles of the Arno River estuary for the two sampling periods.

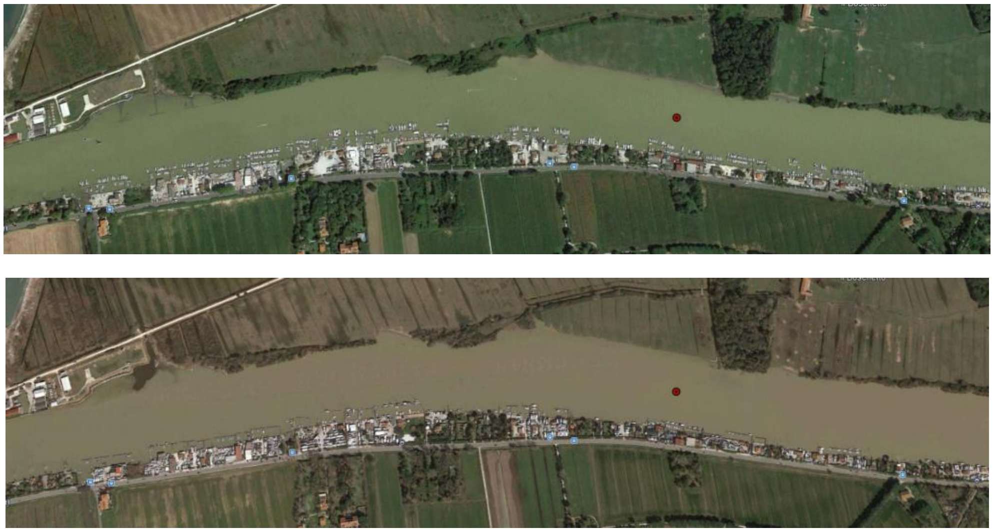

Figure S3. Images taken at the position of E3 site where during summer period numerous boats are anchored (top image; 28.8.2015.) and winter period where boats are mainly located in dry-marina sites (bottom image, 22.3.2018). Dates for google images were chosen to match our sampling seasons (exact dates were not available).

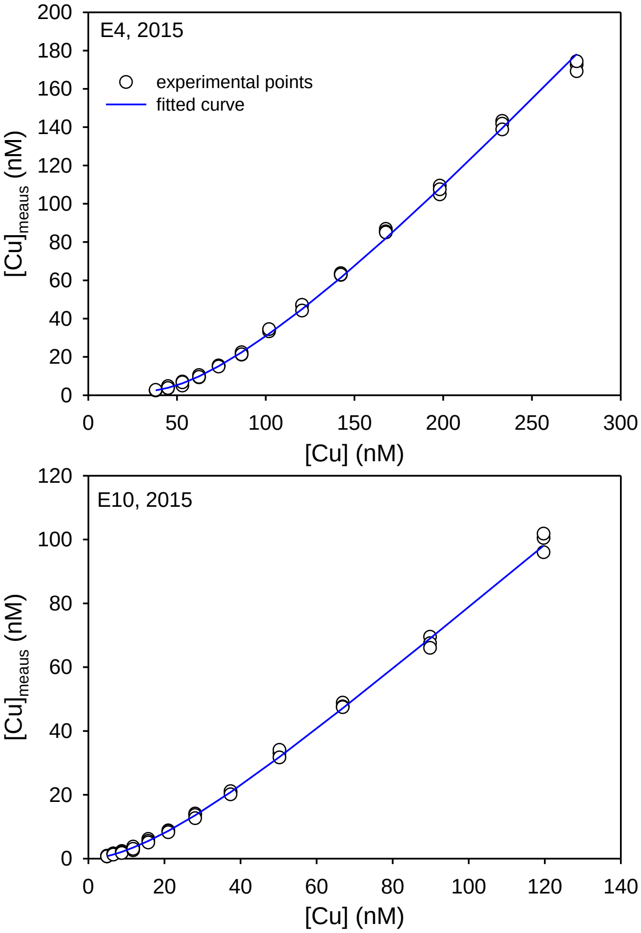

Figure S4. Complexometric titration curves obtained with the addition of 1 mgL^{-1} T-X-100 in E4 and E10 (2015) samples.

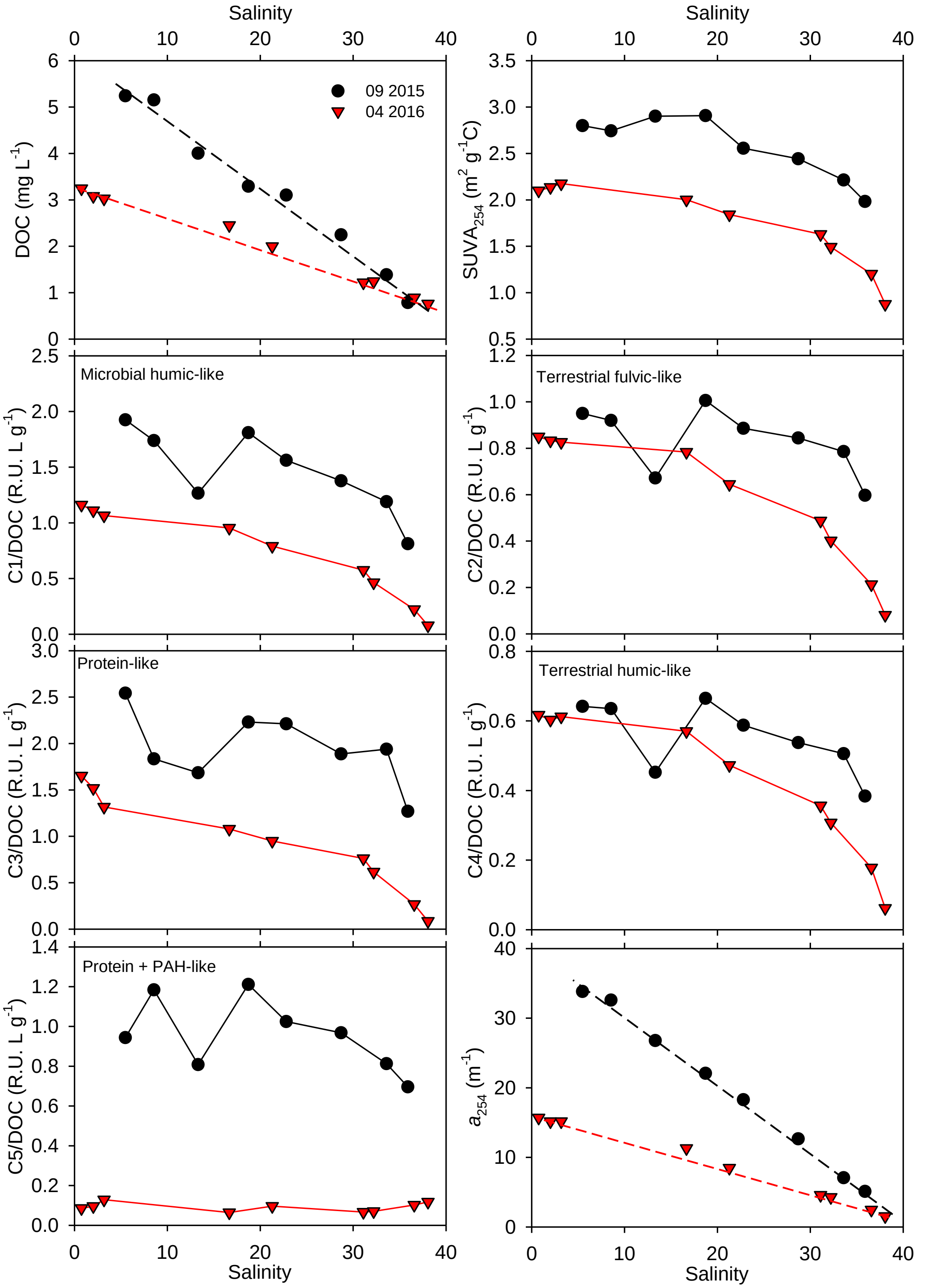

Figure S5. Optical properties of DOM for the two sampling periods. C1-C5 corresponds to components derived by PARAFAC analysis. Dashed lines represent the projected conservative trends.

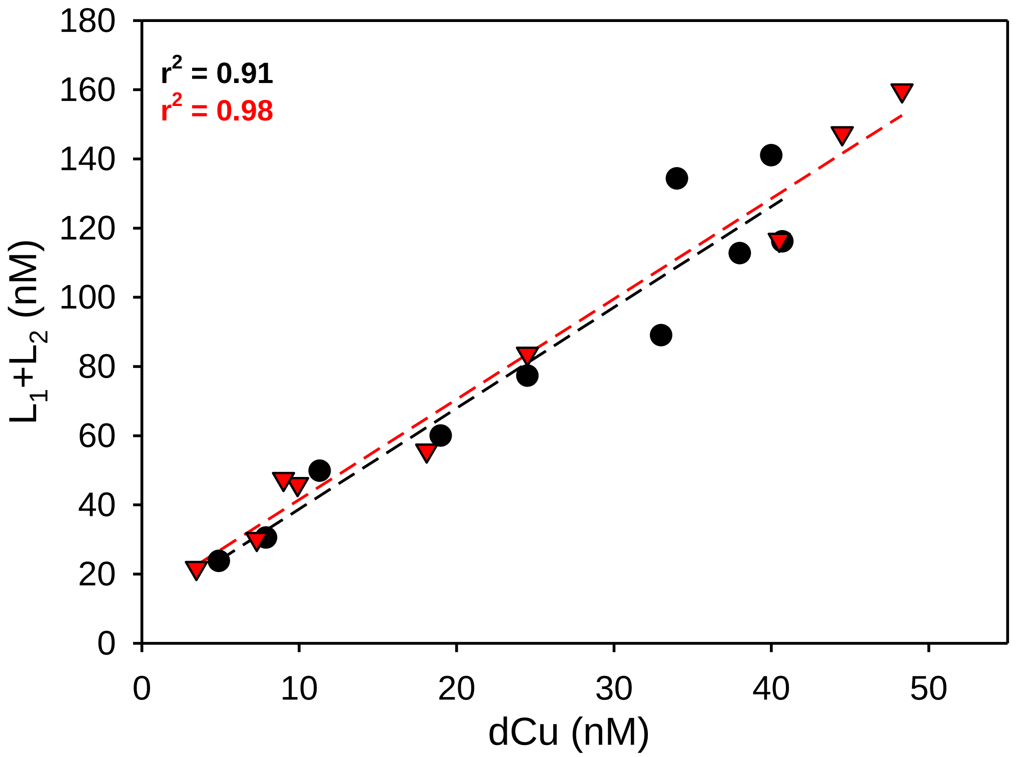

Figure S6. Relationship between the sum of organic ligands (L_1+L_2) and dissolved Cu. Dashed lines represent the linear regression of the data.
